# Remedies for Tooth-Ache

**Published:** 1842-12

**Authors:** 


*Remedies for Tooth-ache.*—Among the ignorant and superstitious of almost every age and nation, the efficacy of charms for the cure, not only of tooth-ache, but for that of nearly every disease incident to the human body, has been believed in. In the surgical work of Ambrose Pare, book 25, chapter 16 and page 605, touching the teeth and repeating these words during mass, "*os non comminuitis ex eo,*" are recommended as "good for the tooth-ache." Repeating the following words are also said, by the same writer, to exert a similar curative effect, "*Strigiles facilisque dentatœ, dentium dolorem perfanate.*"

The curative effects of these remedies, depending upon the contingency that the subject be engaged in saying mass at the time they are had recourse to, we suppose that none but such as belong to that denomination of Christians with whom the observance of the service of mass constitutes a part of their religious worship, would be benefitted by them.

An instance of temporary cure of tooth-ache by a remedy not more potent or rational than either of the preceding, fell under the observation of one of the editors of the Journal, not many years since. The particulars of which are as follows: A countryman called upon him to have a tooth extracted, and this operation having been performed, he immediately jumped from the operating chair, his countenance beaming with joy at the relief it had afforded him, pulled from his bosom a handkerchief, and from it took a paper on which was written in German, a doggerel couplet. This when rendered into English read about as follows:

"Tooth ache, tooth-ache go away,
And from this tooth forever stay."

On inquiry the patient stated that having been attacked about four weeks before with violent tooth-ache, he had applied to a German Charm Doctor, residing somewhere in Pennsylvania, who gave him this scroll, which he directed him to wear in his bosom, assuring him, if he would do so, that it would cure it; and this, he asserted, it did do, for several days, but it having failed to produce the same effect on a recurrence of the pain, he was at last compelled to have recourse to the more certain remedy of extraction.

